# A Retrospective on Nuclear Receptor Regulation of Inflammation: Lessons from GR and PPARs

**DOI:** 10.1155/2011/742785

**Published:** 2011-09-15

**Authors:** Min-Dian Li, Xiaoyong Yang

**Affiliations:** ^1^Department of Cellular and Molecular Physiology, School of Medicine, Yale University, New Haven, CT, USA; ^2^Program in Integrative Cell Signaling and Neurobiology of Metabolism (ICSNM), School of Medicine, Yale University, New Haven, CT 06520, USA; ^3^Section of Comparative Medicine, School of Medicine, Yale University, New Haven, CT 06519, USA

## Abstract

Members of the nuclear receptor superfamily have vital roles in regulating immunity and inflammation. The founding member, glucocorticoid receptor (GR), is the prototype to demonstrate immunomodulation via transrepression of the AP-1 and NF-**κ**B signaling pathways. Peroxisome proliferator-activated receptors (PPARs) have emerged as key regulators of inflammation. This review examines the history and current advances in nuclear receptor regulation of inflammation by the crosstalk with AP-1 and NF-**κ**B signaling, focusing on the roles of GR and PPARs. A better understanding of the molecular mechanism by which nuclear receptors inhibit proinflammatory signaling pathways will enable novel therapies to treat chronic inflammation.

## 1. Introduction

The nuclear receptor (NR) superfamily comprises structurally conserved, ligand-activated transcription regulators that play critical roles in development and homeostasis [1, 2]. In the immune system, it integrates both inflammatory and metabolic signals to maintain homeostasis via positive and negative regulation of gene expression [3, 4]. The immunomodulatory actions of NRs are regulated by ligands such as glucocorticoids, the widely prescribed anti-inflammatory drug [5]. Based on ligands, NRs are grouped into three subfamilies. The first subfamily is the classic endocrine receptors for steroid hormones, thyroid hormones, and vitamin A and D derivatives. The second subfamily is the orphan NRs that share the common structural features of the endocrine receptors, but their ligands have not been identified yet. Over the past decade, a growing number of orphan receptors are “adopted” through the identification of dietary lipids and metabolites as the ligands. These adopted orphan receptors comprise the third subfamily that regulates a wide range of transcriptional programs for tissue homeostasis. 

Depending on ligand availability and promoter context, NRs function both as positive and negative transcriptional regulators. Initially, NRs were thought to be transcriptional activators upon ligand binding [6]. Later studies show that NRs can also repress transcription. The first example is the finding that estrogen receptor (ER) inhibits the prolactin gene when its positive regulatory elements are removed from the promoter [7]. It has now been established that the negative regulation by NRs is crucially important in physiology and diseases [3, 8]. Genome-wide studies show that almost half of ER-target genes are inhibited by estradiol, partially through the association with negative estrogen response elements [9]. Negative glucocorticoid response elements were also well described [8]. Recently, Surjit and colleagues have identified novel negative response elements for ligand-bound GRs, which are responsible for direct repression of over 1000 genes [10]. Additionally, negative regulation by NRs often occurs without direction interaction with *cis* regulatory elements. Instead, NRs can repress transcription via direct interactions with other transcription factors, termed transrepression. 

NR transrepression pathways play a pivotal role in modulating inflammation. Glucocorticoids are widely prescribed drugs to treat autoimmune and inflammatory diseases, and their actions through GR serve as the prototype of NR transrepression. Targets for nonsteroidal anti-inflammatory drugs (NSAIDs), peroxisome proliferator-activated receptors (PPARs), are emerging as key regulators of the immune system [11]. The spectrum of transrepression pathways is expanding. Recently, 5-androgen-3*β*, 17*β*-diol (ADIOL) has been characterized as an endogenous estrogen receptor (ER) *β* ligand to suppress inflammatory responses of microglia and astrocytes by recruitment of CtBP corepressor complexes [12]. Though highly effective in combating both acute and chronic inflammatory diseases, glucocorticoid-based therapy has profound side effects during chronic administration, which is due to the multiple physiological roles of the hormone. For this reason, PPARs have attracted growing attention for drug development. Understanding the molecular details of NR-mediated repression is critical for therapeutic improvement. This paper summarizes the last two decades of research to elucidate the molecular mechanisms of GR and PPAR transrepression pathways and to delineate the crosstalk between these two pathways.

## 2. General Signaling Pathways in Inflammation

Inflammation is a biological response in which the body recruits immune cells to sites of infection, injury, or autoimmune reaction to initiate tissue repair processes [3, 13]. The homeostasis of the immune system is of pivotal importance to human health. Chronic inflammation is strongly associated with a broad range of pathological conditions, such as rheumatoid arthritis, inflammatory bowel diseases, asthma, diabetes, and atherosclerosis.

Activator protein-1 (AP-1) and nuclear factor-*κ*B (NF-*κ*B) are among master regulators of inflammation. They respond to a remarkable variety of external and internal stimuli and control the expression of a diverse array of genes involved in inflammation, cell proliferation, differentiation, and survival [14–16].

**Figure 1:**
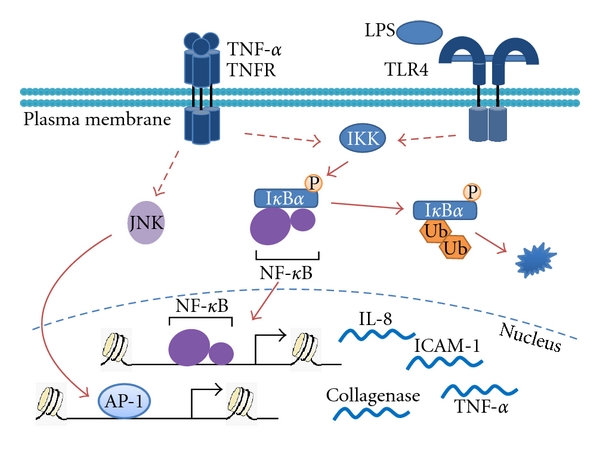
Transcriptional control of inflammation. Signal transduction of proinflammatory cytokines, for example, TNF-*α* and/or LPS signals lead to activation of IKK complex to liberate cytosolic NF-*κ*B from inhibition via ubiquitination and degradation of I*κ*B*α.* These stimuli activate the JNK-AP-1 pathway. Coordinated actions of NF-*κ*B and AP-1 propagate inflammation via promoting transcription of cytokines, chemokines, and other proinflammatory genes.

AP-1 is a group of dimeric basic region-leucine zipper (bZIP) proteins that includes four subfamilies: Jun (c-Jun, JunB, and JunD), Fos (c-Fos, FosB, Fra-1, and Fra-2), Maf, and ATF, which recognize either TPA- (12-*O*-tetradecanoylphorbol-13-acetate-) response elements or cAMP-response elements (CRE) [16]. Depending on cell types, the major form of cellular AP-1 is either the Jun-Fos heterodimer or the Jun-Jun homodimer. The AP-1 signaling pathway is regulated at several levels: first, regulation of *Jun *and *Fos* transcription and mRNA turnover; second, regulation of Jun and Fos protein turnover; third, posttranslational modifications of Jun and Fos proteins that modulate their transcription activity; fourth, recruitment of other proteins that can either synergize or interfere with AP-1 activity, as exemplified by GR [17, 18]. The transcription of the Jun and Fos family genes can be stimulated by cytokines or other physiological signals in an MAP kinase-dependent manner [19, 20] (Figure 1). Jun and Fos then form the heterodimer to activate or repress their target genes. 

The NF-*κ*B transcription factor family in mammals consists of five protein subunits, p65 (RelA), RelB, c-Rel, p105/p50 (NF-*κ*B1), and p100/p52 (NF-*κ*B2). These subunits form hetero- or homodimeric transcription complexes with distinct activities [14]. The p65 (RelA)/p50 heterodimer represents the most abundant form of NF-*κ*B [14, 15]. In quiescent cells, NF-*κ*B retains in the cytoplasm by binding to the inhibitor of *κ*B (I*κ*B) family proteins (I*κ*B*α*, I*κ*B*β*, and I*κ*B*ε*) or the precursor Rel proteins (p105 and p100) [14]. A great variety of stimuli, including proinflammatory cytokines and bacterial endotoxin lipopolysaccharide (LPS), activate the heterotrimeric IKK (I*κ*B kinase) complex, which serves as a critical node that integrates diverse upstream signals. Lysine (K63)-linked and/or the carboxy-terminal glycine (G76)-linked linear polyubiquitination of IKK*γ* (also known as NEMO, NF-*κ*B essential modulator) promotes phosphorylation of the complex, leading to either phosphorylation, polyubiquitination and subsequent proteasomal degradation of I*κ*Bs, or proteolytic processing of p100 into p52 [21, 22]. Consequently, NF-*κ*B is released from inhibition and mobilized to the nucleus (Figure 1). In addition to protein processing of inhibitory modules, posttranslational modifications of the p65 subunit also modulate release and nuclear translocation of NF-*κ*B [21]. Of note, following I*κ*B*α* degradation, phosphorylation of p65 at S276 regulates DNA binding, dimerization, and recruitment of p300/CBP (CREB-binding protein) coactivator complexes [21, 23]. Acetylation of p65, probably catalyzed by p300/CBP or other lysine acetylases, enhances transcriptional activity [24]. Nuclear NF-*κ*B binds directly to and activates target genes in concert with other transcription factors [25, 26]. 

The termination of NF-*κ*B signaling is controlled by multiple mechanisms. NF-*κ*B induces expression of inhibitory proteins (such as I*κ*B*α* and A20) and a subset of microRNA species, which in turn inhibit NF-*κ*B expression or activity [14, 27]. Single-cell studies indicate that negative feedback inhibition by I*κ*B*α* does not terminate the signaling abruptly but generates cyclic presence of NF-*κ*B in the nucleus [28]. Another negative feedback loop is that induction of the deubiquitinase A20 leads to the inactivation of IKK [29]. Positive feedback loops are important for robust oscillation of NF-*κ*B signaling. TNF-*α* cannot only initiate NF-*κ*B signaling, but also promote a secondary wave of NF-*κ*B responses induced by LPS-TLR4 signaling [30], therefore producing positive feedback. In addition to the feedback transcriptional regulation, posttranslational modifications of NF-*κ*B also contribute to temporospatial regulation. For example, deacetylation of p65 by histone deacetylase 3 (HDAC3) promotes the interaction between nuclear I*κ*B*α* and NF-*κ*B, resulting in nuclear export of the complex [31].

 Inflammation is under the combinatorial transcriptional control of NF-*κ*B and AP-1 signaling pathways. Proinflammatory cytokines, such as tumor necrosis factor-*α* (TNF-*α*) and interleukin-1*β* (IL-1*β*), induce AP-1 signaling via MAPK cascades and activate NF-*κ*B signaling by ubiquitination and degradation of I*κ*B*α* [3, 13] (Figure 1). AP-1 and NF-*κ*B coordinate the transcriptional reprogramming of immune cells by stimulating expression of proinflammatory cytokines, chemokines, adhesion molecules, matrix metalloproteases, and others. Sustained inflammation would be detrimental to tissue homeostasis, and multiple mechanisms have evolved to terminate inflammation. Apart from feedback transcriptional and posttranslational regulation mentioned above, a preeminent mechanism is NR-mediated transrepression.

## 3. Molecular Mechanisms of GR Transrepression

### 3.1. Direct Interactions between GR and AP-1

**Figure 2:**
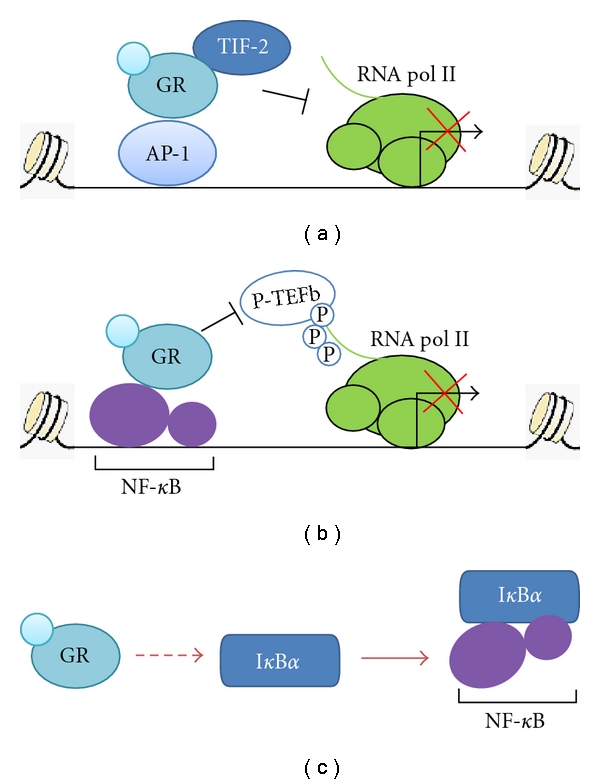
Molecular mechanisms of GR transrepression of AP-1 and NF-*κ*B. (a) Ligand-activated GR is tethered to AP-1 and recruits transcriptional mediators/intermediary factor 2 (TIF-2) to inhibit transcription of AP-1 target genes. (b) Ligand-activated GR bound to NF-*κ*B interferes with recruitment of NF-*κ*B Ser2 CTD kinase, positive transcription elongation factor b (P-TEFb), which is required for the transcription of proinflammatory genes. (c) Ligand-activated GR induces synthesis of I*κ*B*α*, thereby blocking NF-*κ*B nuclear translocation.

GR is a prototypical member of the NR superfamily, initially identified as a potent transcription activator [1, 32]. At that time, it was considered that all the physiological effects of GR are mediated through gene induction [6]. This view was challenged a few years later by the discoveries that GR represses transcription of a variety of genes, including proopiomelanocortin (POMC) gene, via negative glucocorticoid response elements (nGREs) [33, 34]. The binding of liganded GR to the nGRE has been implicated in transcriptional repression of only several proinflammatory genes [35]. As discussed below, the suppressive effects of glucocorticoids on inflammation are largely independent of the DNA-binding activity of GR, but via a tethering mechanism referred to as transrepression. The discovery of GR-mediated inhibition of AP-1 transcriptional activity [34, 36, 37] is the first example of transrepression (Figure 2(a)).

Transcription of the collagenase type I gene is stimulated by AP-1 [38, 39] and repressed by liganded GR [40, 41], which was used to explore the molecular mechanism of GR-mediated repression. Schule and his colleagues identified the inhibitory effects of dexamethasone (Dex, a synthetic GR ligand)-activated GR on both synthetic and endogenous promoters containing binding sites for AP-1 in different cell types [34]. The GR inhibition is independent of the glucocorticoid response element (GRE) but strongly associated with AP-1-binding sites. Nevertheless, the authors did not perform mutation analysis of the AP-1 sites to provide the direct evidence for the requirement of AP-1 sites for GR-mediated repression. Mutation analysis of GR identified that both the ligand-binding domain (LBD) and the DNA-binding domain (DBD) are required for repression of AP-1 activity. 

This study is complemented by Jonat et al. showing that cotreatment with Dex nearly abolished TPA induction of collagenase proteins [36]. Transrepression is distinct from transactivation and involves the direct interaction with AP-1. The conclusion is corroborated by a follow-up study reporting that the repression is mediated by GR monomers rather than transcriptional active dimers [42]. Furthermore, the characterization of dimerization-deficient GR (GR^dim/dim^) knock-in mice reveals that transrepression of AP-1 remains intact while transactivation of tyrosine aminotransferase (TAT) is impaired [43]. The *in vitro *evidence for the direct interaction between GR and AP-1 was also reported by Yang-Yen et al. [37]. However, Jonat et al. and Yang-Yen et al. disagreed on whether the DNA binding property of AP-1 is impaired by physical association with activated GR, which might be attributed to their different sources of GR and AP-1 proteins. Human cell lysates show enhanced association between AP-1 and its target DNA sequence probably because of enhanced c-Jun transcription following Dex treatment [36], which is impossible in the *in vitro *assay system. Alternatively, it could be due to the different compositions of AP-1 used in their assays. Yang-Yen et al. used c-Jun monomers, instead of c-Jun/c-Fos heterodimers, in the *in vitro* assay. A follow-up study reported that *in vitro *synthesized GRs do not interfere the binding of c-Jun/c-Fos heterodimers or purified AP-1 *in vitro *[44].

Despite slight discrepancies in the detail, the reports above uniformly unravel a novel mechanism of GR-mediated repression via a direct interaction with a transcription factor, AP-1, but not direct association with DNA. The essential features of crosstalk between NRs and AP-1 signaling pathways seems to be highly conserved, since RAR*α* and TR*α* have also been shown to antagonize AP-1 signaling following the same mechanism [45, 46]. NR-mediated regulation of AP-1 is likely to be dynamic and dependent on the promoter context. Although GRIP-1/TIF-2 is a coactivator for both GR and TR, a study has shown that GRIP-1/TIF-2 can potentiate GR-mediated transrepression of the collagenase-3 gene in human osteosarcoma cells but has no effect on the transrepression by TR [47] (Figure 2(a)).

### 3.2. Direct Interactions between GR and NF-*κ*B

The discovery of GR inhibition of AP-1 sparked the exploration of whether NF-*κ*B is also a target of GR. A few groups reported that, similar to AP-1, activated GR inhibited NF-*κ*B-mediated transcription of proinflammatory genes, including IL-6 and ICAM-1, via direct physical interaction with the p65 subunit of NF-*κ*B [48–50]. Nissen and Yamamoto scrutinized the molecular details of GR inhibition by mapping regions of both GR and p65 that are involved in their association and probing the biochemical composition of RNA polymerase II (pol II) complexes at the promoters of IL-8, ICAM-1 and I*κ*B*α* genes via chromatin immunoprecipitation (ChIP) assays [51]. *In vitro *assays identified that both the DBD and the LBD of GR interact with the dimerization domain of p65. Interestingly, the same regions in GR are involved in the interaction with AP-1, suggesting the existence of a common repression complex and a conserved repression mechanism for AP-1 and NF-*κ*B. The large subunit of RNA polymerase II has a unique carboxyl-terminal domain (CTD) that comprises conserved YSPTSPS heptad repeats. Phosphorylation of the heptad repeats at Ser2 is required for transcription. ChIP data reveal that GR can interfere with Ser2 phosphorylation of pol II CTD at the promoter regions of IL-8 and ICAM-1 genes, whereas neither the binding of NF-*κ*B to DNA nor the assembly of preinitiation complexes is affected under repressing conditions. The phospho-Ser2 level at the I*κ*B*α* promoter is unaffected by Dex. Thus, GR represses NF-*κ*B-stimulated transcription of a subset of inflammatory genes by suppressing pol II CTD phosphorylation. Given that neither HDAC recruitment nor putative Ser2 phosphatase has been identified, it will be important to uncover the identity of corepressors of GR inhibition.

There are several different but not mutually exclusive explanations for GR-induced transrepression of NF-*κ*B signaling (Figure 2(b)). First, GR can antagonize with protein kinases that modify pol II CTD. Luecke and Yamamoto reported that GR prevents the recruitment of the Ser2 CTD kinase complex P-TEFb (positive transcription elongation factor b) to the promoter of IL-8 but not I*κ*B*α*, probably by interfering the physical interaction between NF-*κ*B and P-TEFb [52]. Second, ligand-bound GR is likely to facilitate another posttranslational modification that competes with phosphorylation of CTD [53]. Third, the specificity of GR transrepression can be attributed to the composition of NF-*κ*B activation complexes. Activated GR also disrupts the NF-*κ*B/interferon regulatory factor (IRF) enhanceosomes that are responsible for activation of a large set of TLR4- and TLR9-dependent inflammatory genes [54, 55].

### 3.3. Regulation of I*κ*B by GR

Despite substantial evidence to support the transrepression mechanism, it should be cautious to make a sweeping conclusion that this mechanism accounts for all the inhibitory effects of GR on NF-*κ*B. The studies from Baldwin's and Karin's groups suggest that glucocorticoids modulate NF-*κ*B signaling by stimulating transcription of the gene encoding the NF-*κ*B inhibitor protein—I*κ*B [56, 57] (Figure 2(c)). Auphan and colleagues have shown that glucocorticoids are potent inhibitors of NF-*κ*B activity in mouse immune organs and several lymphoma cells [57]. Dex can abolish NF-*κ*B DNA-binding activity through GR in human lymphoma Jurkat cells. Additionally, Dex-activated GR also induces the synthesis of I*κ*B*α*, resulting in the sequestration of NF-*κ*B in the cytoplasma. The involvement of protein synthesis in the inhibition of NF-*κ*B signaling argues against direct interaction between GR and NF-*κ*B in lymphoma cell lines. 

However, GR-induced synthesis of I*κ*B*α* is dispensable for transrepression of NF-*κ*B signaling. Several studies have demonstrated that transrepression and transactivation properties of GR can be separated [42, 58]. A dimerization-defective mutant of human GR that fails to induce I*κ*B*α* expression can effectively inhibit transcriptional activity of NF-*κ*B. Moreover, some glucocorticoid analogs can enhance the synthesis of I*κ*B*α* but fail to repress NF-*κ*B activity. The first *in vivo *evidence came from further characterization of the dimerization-deficient mutant GR (A458T) knock-in mouse model which had been used to demonstrate that GR transrepresses AP-1 signaling despite loss of transactivation *in vivo* [43, 59]. GR (A458T) can effectively repress both local and systemic inflammatory responses via repressing NF-*κ*B in the absence of DNA binding. Nevertheless, studies using GR DBD mutants suggest that GR-mediated transrepression of AP-1 and NF-*κ*B signaling may involve different mechanisms [60]. A point mutation in the second zinc finger of DBD (R488Q) abolishes the ability of GR to repress a subset of NF-*κ*B target genes but not AP-1-luciferase report activity.

## 4. Molecular Mechanisms of PPAR Transrepression

PPARs are adopted NRs that modulate metabolism and inflammation [3, 61]. There are three types of PPAR isoforms: *α*, *δ/β*, and *γ*, with distinct biological functions. Previous studies have uncovered multiple mechanisms by which PPARs suppress proinflammatory gene expression, including the inhibition of NF-*κ*B and AP-1 signaling pathways and the retention of corepressor complexes.

### 4.1. PPAR Inhibition on AP-1 and NF-*κ*B

It has been reported that PPARs suppress inflammation by inhibiting the activity of other transcription factors, including AP-1 and NF-*κ*B [11]. For example, PPAR*α* inhibits expression of IL-6, prostaglandin, and cyclooxygenase-2 (COX-2) via repression of NF-*κ*B signaling in aortic smooth muscle cells, thus possibly reducing the risk for atherosclerosis [62]. PPAR*γ* can attenuate macrophage activity via antagonizing AP-1, NF-*κ*B, and STAT1, as revealed by transcription reporter assays [63]. 

**Figure 3:**
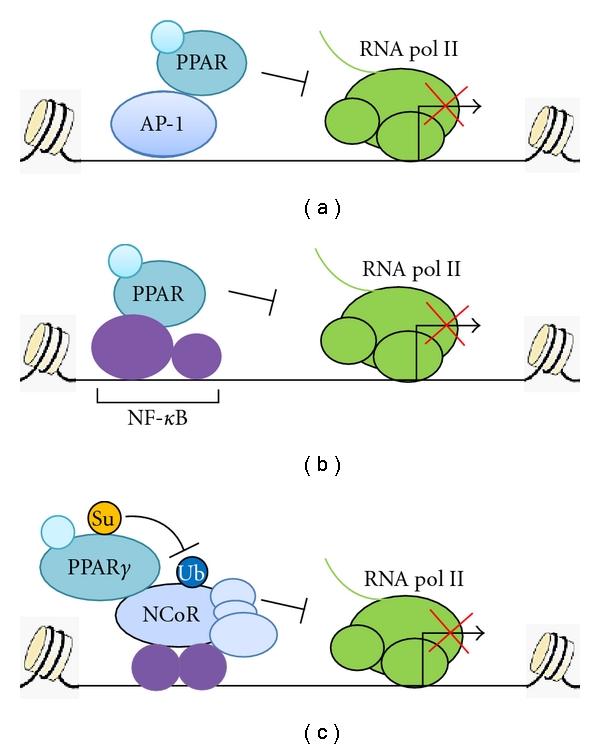
Molecular mechanisms of PPAR transrepression pathways. (a-b) Ligand-activated PPAR (*α* or *γ*) inhibits the activities of AP-1 and NF-*κ*B through direct interactions with the c-Jun subunit and the p65 subunit, respectively. (c) On ligand binding, PPAR*γ* is sumoylated and blocks ubiquitin-dependent proteasomal degradation of the NCoR corepressor complexes at NF-*κ*B-binding sites. The presence of the corepressor complexes prevents the recruitment of transcriptionally active NF-*κ*B, leading to inhibition of inflammatory gene expression.

Delerive and colleagues characterized direct interactions of PPAR*α* with AP-1 and NF-*κ*B [64] (Figures 3(a)-3(b)). PPAR*α* inhibits vascular inflammation in arotic smooth muscle cells by physical interactions with c-Jun and p65. Interestingly, the regions of c-Jun and p65 that bind to PPAR*α* also interact with GR. On the other hand, the synthetic PPAR*α* ligand called fibrate can induce the expression of I*κ*B*α* in both smooth muscle cells and hepatocytes, resulting in sequestration of NF-*κ*B in the cytoplasma and reduction in its DNA-binding ability. These findings are corroborated by the results from PPAR*α*-null mice, showing that the induction of I*κ*B*α* expression is PPAR*α*-dependent [65]. Therefore, PPAR*α* and GR appear to share similar mechanisms to repress AP-1 and NF-*κ*B, respectively.

PPAR*γ* transrepression pathways also impinge on AP-1 and NF-*κ*B. PPAR*γ* has been shown to inhibit AP-1 association with DNA and activation in vascular endothelial cells and lungs, respectively [66, 67]. Modulated by physiological ligands from oxidized low-density lipoproteins (oxLDL), PPAR*γ* can also inhibit interleukin-12 (IL-12) production in marcophages through direct interaction with NF-*κ*B [68].

### 4.2. PPAR*γ* and Sumoylation-Dependent Association with Corepressor Complexes

In addition to transrepressing specific transcriptional activators, PPARs have been demonstrated to prevent the clearance of corepressor complexes at the promoter regions (Figure 3(c)). The NR corepressor (NCoR)/SMRT-HDAC3 corepressor complex is recruited by several unliganded NRs to mediate transcriptional repression [69–71]. Recent data show that NCoR/SMRT-HDAC3 corepressor complexes are also required for basal repression of a subset of AP-1 and NF-*κ*B target genes through association with inhibitory homodimers (cJun-cJun and p50-p50), respectively [72–74]. Using the inducible nitric oxide synthase (*iNOS*) gene in mouse macrophages as a model, Pascual and colleagues uncovered a novel transrepression pathway that PPAR*γ* represses this NF-*κ*B target gene via association with NCoR, leading to stabilization of the corepressor complexes [75]. Ligand-dependent activation of PPAR*γ* is associated with SUMOylation of the ligand-binding domain (LBD), which promotes the physical interaction between PPAR*γ* and NCoR. This direct interaction targets the NR to the NCoR-HDAC3 corepressor complexes at proinflammatory gene promoters and thus prevents LPS-induced recruitment of the ubiquitination/19S proteosome machinery to remove the corepressor complexes. Consequently, the impaired clearance of the NCoR/SMRT-HDAC3 complexes blocks the exchange of repressive homodimers for active heterodimers of NF-*κ*B [72]. The corepressor complexes are also involved in the regulation of AP-1 target genes in macrophages [73], which is probably influenced by this sumoylation-dependent transpression pathway. Likewise, liver X receptors (LXRs) also adopt this transrepression mechanism [76]. Taken together, these studies delineate a molecular pathway featured on NR-mediated stabilization of corepressor complexes.

## 5. Crosstalk between PPARs and GR Transrepression Pathways

The extensive crosstalk between PPARs and GR in immunomodulation has emerged as a key strategy to combat chronic inflammatory diseases [54, 77]. Genome-wide gene expression profiling data shows that GR and PPAR*γ* function in a combinatorial manner to repress LPS-responsive genes [54], indicating differential transrepression pathways for these two NRs (see Sections 3.2 and 4.2). In contrast, PPAR*α* and GR seem to share several common features of NF-*κ*B and AP-1 inhibition. Simultaneous activation of both NRs lead to corepression of NF-*κ*B target genes [77]. PPAR*α* can directly associate with GR. This interaction enhances GR transrepression and, at the same time, blocks the recruitment of GR to glucocorticoid-responsive elements and thus inhibits transactivation of GR target genes. The unexpected finding that PPAR*α* can prevent the GR-mediated transactivation implies that coadministration of glucocorticoids and PPAR*α* ligands can enhance the immune-modulatory effects and reduce the side effects caused by glucocorticoids [77, 78].

## 6. Conclusions and Future Perspectives

Decades of research have characterized multiple molecular pathways of NR-mediated negative regulation of inflammatory genes. In this framework, on binding to their specific ligands, NRs have at least two different, but not mutually exclusive, mechanisms to inhibit transcription. First, NRs can directly inhibit the activities of NF-*κ*B and AP-1. In addition, combinatorial actions of different NRs can optimize both the strength and specificity of transrepression. For example, PPAR*α* and GR can function synergistically to repress the expression of inflammatory genes as well as transaction activity of their own [77]. Second, NRs can induce the expression of genes that inhibit inflammatory signaling pathways. Transrepression of AP-1 and NF-*κ*B signaling pathways results in decrease in cytokine production and other proinflammatory responses, bringing the immune system back to homeostasis. 

However, numerous questions remain to be addressed. First, we have focused on several NR transrepression pathways to illustrate some general principles, but the composition and dynamics of the underlying signaling circuit have yet to be fully uncovered. In addition to discovering novel mechanisms, further studies should delineate the relationship among existing pathways. For example, PPAR*γ* probably also interferes with phosphorylation of the CTD of RNA polymerase II; likewise, GR and PPAR*α* are possible to stabilize corepressor complexes associated with inflammatory genes. Several studies have implied that corepressor complexes can function as a checkpoint for transcriptional control [73, 75]. Here, we would elaborate on the checkpoint by proposing the following model: Ligand-bound NRs are targeted to inflammatory genes by activated NF-*κ*B or AP-1 and then halt transcription at the checkpoint by (1) stabilizing NCoR/SMRT-HDAC corepressor complexes, (2) preventing association of active transcription factors and coactivators, (3) maintaining the inhibitory histone modifications, and (4) modifying the CTD of RNA polymerase II. The completion of all these molecular events may contribute to transrepression of inflammatory genes.

Second, given evidence that different NRs target different subsets of inflammatory genes [54], it will be important to define these subgroups and assess the overlapping function of NRs. In addition, the transrepression pathways exhibit tissue specificity, such as different GR responses in immune and nonimmune cells. Recent advances by Cidlowski's group and others have shown that multiple isoforms of GR can be generated from the sole GR gene via alternative splicing and selective translational initiation, which exhibit tissue-specific distribution and different regulatory mechanisms [79]. NRs also recruit different coregulators and/or other transcription factors to target specific gene sets, as inferred from current studies. The identification of these NR isoforms, co-regulators, and histone modifications is not only important for understanding NR regulation of inflammation, but also beneficial for therapeutic intervention of inflammation.

Third, glucocorticoids are released from adrenal glands in circadian and ultradian modes to modulate inflammatory responses. Ultradian hormone release stimulates cyclic GR-mediated transcriptional regulation [80]. Whether this temporal regulation might be relevant to GR-mediated transrepression, whether this could be observed in other endocrine or adopted NRs, and whether pulse administration can improve current clinical protocol are poorly studied. A better understanding of temporal regulation of NR transrepression pathways and their physiological significance will bring profound clinical benefits.

Finally, chronic inflammation is associated with various metabolic diseases, such as cardiovascular diseases, type II diabetes, and obesity [81, 82]. Synthetic NR ligands have been widely used to control chronic inflammation. As dietary lipid and metabolites can serve as endogenous NR ligands, an interesting question is whether the body's metabolic state impinges on the immune system via NR signaling. 

In closing, inflammation is integral to a complex system that maintains the body's homeostasis. Unraveling temporal and spatial regulation of inflammation by NRs using a combination of biochemical, genetic, genomic, and proteomic tools will aid in the design of novel therapies for inflammatory diseases.
